# The Use of Physiological Signals in Brainstem/Midbrain fMRI

**DOI:** 10.3389/fnins.2018.00718

**Published:** 2018-10-16

**Authors:** Andy Schumann, Stefanie Köhler, Feliberto de la Cruz, Daniel Güllmar, Jürgen R. Reichenbach, Gerd Wagner, Karl-Jürgen Bär

**Affiliations:** ^1^Psychiatric Brain and Body Research Group Jena, Department of Psychiatry and Psychotherapy, Jena University Hospital, Jena, Germany; ^2^Medical Physics Group, Institute of Diagnostic and Interventional Radiology, Jena University Hospital, Jena, Germany; ^3^Michael Stifel Center for Data-driven and Simulation Science Jena, Friedrich Schiller University Jena, Jena, Germany

**Keywords:** locus coeruleus, Stroop task, substantia nigra, pupil diameter, skin conductance

## Abstract

Brainstem and midbrain nuclei are closely linked to cognitive performance and autonomic function. To advance the localization in this area, precise functional imaging is fundamental. In this study, we used a sophisticated fMRI technique as well as physiological recordings to investigate the involvement of brainstem/midbrain nuclei in cognitive control during a Stroop task. The temporal signal-to-noise ratio (tSNR) increased due to physiological noise correction (PNC) especially in regions adjacent to arteries and cerebrospinal fluid. Within the brainstem/cerebellum template an average tSNR of 68 ± 16 was achieved after the simultaneous application of a high-resolution fMRI, specialized co-registration, and PNC. The analysis of PNC data revealed an activation of the substantia nigra in the Stroop interference contrast whereas no significant results were obtained in the midbrain or brainstem when analyzing uncorrected data. Additionally, we found that pupil size indicated the level of cognitive effort. The Stroop interference effect on pupillary responses was correlated to the effect on reaction times (*R*^2^ = 0.464, *p* < 0.05). When Stroop stimuli were modulated by pupillary responses, we observed a significant activation of the LC in the Stroop interference contrast. Thus, we demonstrated the beneficial effect of PNC on data quality and statistical results when analyzing neuronal responses to a cognitive task. Parametric modulation of task events with pupillary responses improved the model of LC BOLD activations in the Stroop interference contrast.

## Introduction

Accumulating research has revealed the close interrelationship between the autonomic state and motivation, attention, mood, or cognition. For instance, in addition to the strong influence of cognitive strains on autonomic function, feelings in the body that are associated with emotions (somatic markers) profoundly influence our decisions (Damasio et al., 1991; Bechara and Damasio, 2005). Thus, the internal state determines the way we react to the environment (Critchley, 2009). It has been assumed that behavioral states might be related closely to neuromodulatory brainstem systems. Thus, assessing the activity of the autonomic nervous system is indicative of the functional state (Samuels and Szabadi, 2008b; Urai et al., 2017). Therefore, inclusion of peripheral indicators of autonomic function is an important addendum to research in neurosciences.

One excellent example of such a close interaction is the human pupil reflecting both the autonomic state as well as cognitive demand (Granholm and Steinhauer, 2009; Laeng et al., 2012). Thus, pupillometry has become a powerful tool in psychophysiology (e.g., see review by Sirois and Brisson, 2014). Pupils have been shown to react to various types of cognitive demand, such as memory load (Attar et al., 2013), arithmetic tests (Võ et al., 2008), or speech processing (Zekveld et al., 2010). A higher level of cognitive effort leads to a greater magnitude of pupillary reaction (Laeng et al., 2012; Querino et al., 2015). The size of the pupil is determined by the sympathetic and parasympathetic outflow to the muscles of the iris (Steinhauer et al., 2004). Parasympathetic activation leads to constriction, whereas sympathetic stimulation results in dilation of the pupil. Preganglionic sympathetic neurons receive input from the posterior nuclei of the hypothalamus and project from the intermediolateral nucleus in the spinal cord through the sympathetic trunk to the iris. The Edinger-Westphal nucleus (EWN) is considered to be the primary origin of parasympathetic influence on the pupils. In addition, the locus coeruleus (LC), the main source of noradrenaline in the brain, exhibits inhibitory influences on the EWN (Samuels and Szabadi, 2008a; Murphy et al., 2014; Joshi et al., 2016). Given the reported co-variation of LC firing rate and pupillary responses (Aston-Jones and Cohen, 2005; Costa and Rudebeck, 2016), the dilation of the pupil has been considered as a proxy of noradrenergic activity in humans (Sirois and Brisson, 2014; Eckstein et al., 2017).

The LC has been proposed to facilitate the dynamic reorganization of neural networks in response to external stimuli (Bouret and Sara, 2005). Corbetta et al. (2008) highlighted its role in attention shifting and cognitive flexibility. They postulated that the LC is significantly involved in the dynamic switch between functional networks. The dorsal fronto-parietal network including the dorsolateral prefrontal cortex and the dorsal parietal cortex is important for the selection of stimuli and generation of responses. The ventral fronto-parietal network with the temporo-parietal junction, ventrolateral prefrontal cortex and anterior insula as core regions, detects salient stimuli, interrupts and redirects attention (Corbetta et al., 2008).

Another physiological marker that has been proposed to indicate mental effort is the level of skin conductance (Jacobs et al., 1994; Kohlisch and Schaefer, 1996; Mehler et al., 2009; Reimer and Mehler, 2011). The secretion of sweat changes the electrical properties of the skin that can be recorded superficially (Edelberg, 1993). In competitive analyses, Kahneman et al. (1969) and Fritz et al. (2014) demonstrated measures of skin conductance and pupil size to be similarly suitable for indicating mental workload. Hogervorst et al. (2014) reported the pupil size to be more accurate than skin conductance to reflect cognitive load.

The differential involvement of neurotransmitter systems in the generation of skin conductance responses (SCR) is still unclear. Yamamoto et al. (1990) showed that lesions to noradrenergic fiber bundles in cats abolish SCR and spontaneous fluctuation of skin conductance. In contrast, microinjections into the ventral tegmental area (VTA), the midbrain’s dopaminergic center, had no effect on skin conductance. Neuroimaging studies reported that BOLD changes and metabolism in regions congruent to LC and VTA are related to spontaneous skin conductance fluctuations at rest (Patterson, 2002), and responses to fear (Linnman et al., 2013). Sequeira and Roy (1993) proposed a crucial influence of the reticular formation, a brainstem network including the LC, on skin conductance. Thus, the noradrenergic system might have a decisive role in generating SCR.

Recently, we reported that the LC and VTA/Substantia nigra (SN) are significantly involved in cognitive and especially inhibitory control (Köhler et al., 2016, 2018b). We used the well-established *Stroop Color-Word* Test [SCWT, (Stroop, 1935)] to investigate the neural correlates of cognitive control processes in the brainstem/midbrain. In the SCWT, participants shall name the color in which a word is presented ignoring the word itself. The color either matches the meaning of the word (congruent condition) or not (incongruent condition, IC). Incongruent trials require an inhibition of an overlearned, pre-potent response tendency (reading the word) in favor of an unusual, less pre-potent action (naming the color of the ink). We observed that BOLD activation of the LC and VTA/SN was higher in incongruent than in congruent trials. Compared to the VTA/SN, the LC was functionally connected to the dorsal fronto-parietal network. We additionally demonstrated differential activation of VTA/SN and LC modulated by demands of cognitive control. Our data gave additional evidence that phasic activation of LC facilitates cognitive conflict resolution and optimizes behavioral responses by activating specific task-related brain networks.

However, brainstem and midbrain nuclei are small compared with the cortical regions involved in cognitive control, and are more susceptible to signal distortion and artifacts arising from local tissue interfaces and physiological noise (Köhler et al., 2016). Especially the position of the LC at the posterior border of the brainstem impedes its functional imaging. Neuromelanin-sensitive scans have added significant information on the anatomical location of the LC (Keren et al., 2009, 2015; Hämmerer et al., 2017) that can be used to validate the identification of LC (Astafiev et al., 2010; Murphy et al., 2014; Clewett et al., 2018). Standard anatomical scans can also be used to detect activation of the LC (Brooks et al., 2017; de Gee et al., 2017). To link structures such as the LC with specific functions using fMRI, a precise match of anatomical and functional information is essential. To really advance our understanding in this field, improvement of functional data analysis is needed.

In our present study, we used the SCWT to corroborate the involvement of LC and VTA/SN in cognitive control. To further enhance image quality, we prepared our data by applying physiological noise correction (PNC) and a specialized brainstem/cerebellum normalization technique. To improve anatomical and functional distinction between brainstem and midbrain nuclei, we applied functional scans with a high spatial resolution of 1.4 mm^3^ and assessed simultaneously physiological responses of pupils and skin conductance to indicate cognitive efforts. We assumed a close association of LC activation and pupillary dilation in the Stroop interference contrast. Therefore, we hypothesize that including pupillary responses to model BOLD activation within the LC might enhance its identification.

## Materials and Methods

### Subjects

Fourteen healthy subjects (nine females, five males, age: 27 ± 7 years) participated in this study. The following exclusion criteria were applied: any disease or impairment of the circulatory system, the peripheral nervous system or the endocrine system, alcohol- or drug abuse. Subjects with past or current neurological or psychiatric diseases according to M.I.N.I (Sheehan et al., 1998) and/or first-degree relatives with Axis I psychiatric disorders were excluded from the study. None of the study participants was taking any psychopharmacological medication.

Three subjects were excluded due to inadequate quality of the eye tracker signal (see below). Thus, eleven subjects were analyzed (seven males, four females, age: 28 ± 7 years). All participants were German native speakers, right-handed according to the modified version of Annett’s Handedness Inventory (Briggs and Nebes, 1975). All participants gave written consent to a protocol approved by the Ethics Committee of the University Hospital Jena in accordance with the Declaration of Helsinki.

### Experimental Paradigm

Participants conducted the manual version of the Stroop color word task (Stroop, 1935; Wagner et al., 2015). In brief, participants had to choose the color of the word written in the center of the projected screen. In the congruent condition, the color of the word matches its meaning. In the IC, the color word is displayed in a color, which is not denoted by the word. Subjects were instructed to indicate one of two possible answers presented at the bottom of the screen by pressing a button of the pad with the right index or middle finger. Correct answers were counterbalanced on the right and left sides of the display. In a pseudorandomized order, 18 congruent and 18 incongruent stimuli were presented for 1500 ms each, with an inter-stimulus interval of 10.5 s. Additionally, a temporal jitter was introduced to enhance the temporal resolution.

### MRI Parameters

Functional data was collected on a 3 T Magnetom PRISMA fit whole-body system (Siemens Healthineers, Erlangen, Germany) equipped with a 64-element receive-only head matrix coil. Head immobilization was achieved using head pads within the coil. T2^∗^-weighted images were obtained using a gradient-echo EPI sequence (TR = 2040 ms, TE = 33 ms, flip angle = 75) with 100 contiguous transverse slices of 1.4 mm thickness and a multi-band acceleration factor of four covering the entire brain and including the lower brainstem. Matrix size was 138 × 138 pixels with in-plane resolution of (1.4 × 1.4) mm^2^. A series of 220 whole-brain volume sets were acquired in one session. High-resolution anatomical T1-weighted volume scans (MP-RAGE) were obtained in sagittal orientation (TR = 2300 ms, TE = 2.07 ms, TI = 900 ms, flip angle = 9%, FOV = 256 mm, matrix = (256 × 256) mm^2^, number of sagittal slices = 192, with an isotropic resolution of (1 × 1 × 1) mm^3^.

### Physiological Recordings

Photoplethysmogram (PPG), respiration and skin conductance were recorded throughout MR image acquisition at 500 Hz using an MR-compatible polygraph MP150 (BIOPAC Systems Inc., Goleta, CA, United States). Respiratory activity was assessed by a strain gage transducer incorporated in a belt that was tied around the chest, approximately at the level of the processus xiphoideus. The respiratory signal was amplified between 0.05 and 10 Hz and temporally smoothed (250 samples). The PPG sensor was attached to the proximal phalanx of the left index finger. The finger pulse signal was recorded in a frequency range of 0.05–3 Hz and smoothed over 50 samples. Skin conductance was measured continuously (constant voltage 1technique) at the left hand’s palm with Ag/AgCl electrodes placed at the thenar and hypothenar eminence. The signal was amplified below 10 Hz, median filtered (150 samples) and smoothed (250 samples).

Pupillometric recordings were conducted using a MR-compatible ASL Long Range Optic Eye Tracker (Applied Science Laboratories, Bedford, MA, United States). Pupil diameter (PD) and gaze position were extracted from the video stream and sampled at 120 Hz. Artifacts and closed eyes were automatically detected by the software and replaced offline by linear interpolation of adjacent values. PD was smoothed over 100 samples. Only recordings with artificial samples less than 10% of all acquired data during the recording were analyzed (mean artifact rate of the final sample was 3.3%). Trigger output of the MR scanner was logged by the presentation software and recorded by the polygraph and the eye tracker to synchronize all the acquired data with the task paradigm.

### Physiological Noise Correction

Prior to preprocessing, physiological fluctuations synchronized with cardiac and respiratory cycles were removed using the RETROICOR approach (Glover et al., 2000). To account for low frequency variations in the BOLD signal through slow blood oxygenation level fluctuations, five respiration volumes per time (RVT) regressors were additionally removed (Birn et al., 2006). The RVT regressors consisted of the RVT function and four delayed terms at 5, 10, 15, and 20 s (Birn et al., 2008; Jo et al., 2010), while eight low-order Fourier time series (four based on the cardiac phase and four on the respiratory phase) were created using the RETROICOR algorithm. All regressors were generated on a slice-wise basis by AFNI’s RetroTS.m script implemented in MATLAB 2016b, which takes the cardiac and respiratory time series synchronized with the fMRI acquisition as input.

### Image Data Preprocessing

For image processing and statistical analyses, we used SPM12^1^. The first four images were discarded to avoid contributions from non-steady-state tissue magnetization. The remaining 216 images were corrected for differences in time acquisition by sinc-interpolation and realigned to the first image. The co-registered anatomical images were segmented using the tissue probability maps. Functional images were then spatially normalized to the MNI space using parameters estimated during the segmentation process.

### Specialized Brainstem/Midbrain Co-registration

To improve the image normalization at the brainstem level, data was normalized to the spatially unbiased infra-tentorial template [SUIT, version 3.1, (Diedrichsen, 2006)]. Using the SUIT toolbox, we applied the following preprocessing steps: (i) segmentation of the whole-brain image; (ii) cropping of the image, retaining only the cerebellum and brainstem; (iii) normalization using the DARTEL engine (Ashburner, 2007) that uses gray and white matter segmentation maps produced during cerebellar isolation to generate a flow field using Large Deformation Diffeomorphic Metric Mapping (Beg et al., 2005), and (iv) re-slicing to a voxel size of (1.5 × 1.5 × 1.5) mm^3^.

Whole brain and cropped data were smoothed with a Gaussian filter of 4 mm FWHM and high-pass filtered with a cutoff period of 128 s and corrected for serial correlations choosing AR (1).

### Temporal Signal-to-Noise Ratio (tSNR)

The quality of the data was quantified in terms of the temporal signal-to-noise ratio (tSNR). It was calculated voxel-wise as the BOLD signals temporal mean divided by its standard deviation (Brooks et al., 2013). To compare the change of quality due to PNC at the brainstem level, we calculated mean tSNR maps after standard preprocessing of corrected and uncorrected data (standard co-registration, unsmoothed). Additionally, we estimated a mean tSNR map of the data used for our analyses (with specialized co-registration, PNC and smoothing).

### Physiological Recordings: Event-Related SCR and PDR

Physiological responses were extracted at the onset of stimulus presentation (reference time *t* = 0) with a duration of 10 s and referenced to baseline (mean value within 1 s before stimulus onset). We averaged reactions in congruent and incongruent Stroop trials. The area under the curve (AUC) and maximum of each PD and SC reaction was estimated and compared in terms of a paired *t*-test between conditions. The AUC per subject was used as measure of the strength of PD and SCR in all further analyses. The pupillary Stroop effect was defined as percentage increases of PDR in IC compared to CC (Rondeel et al., 2015). Stroop interference effect, i.e., the percentage increase of reaction times from CC to IC was correlated to the pupillary Stroop effect.

### FMRI Statistical Data Analysis

Reaction times and characteristics of physiological responses to congruent and incongruent Stroop stimuli were compared by a paired *t*-test. To assess a possible decline of physiological reactions over trials, we standardized all reactions (AUC) to the first trial and correlated these ratios to the trial number.

In *model 1*, neural responses to incongruent (IC) and congruent Stroop stimuli (CC) were modeled by convolving a series of impulses at stimulus onset times by the hemodynamic response function. The single-subject general linear model (GLM) included the regressors of CC and IC, as well as six head motion parameters estimated during image realignment.

In *model 2*, each Stroop stimulus was weighted by the evoked SCR. Therefore, SCR was added as a parametric modulator to *model 1*. Single subjects GLMs included modulated task regressors (CC and IC) and six head motion parameters as covariates.

In *model 3*, pupillary responses were used for parametric modulation of Stroop stimuli in the same manner as in *model 2*.

Subject-specific parameter estimates were then entered into a second-level RFX analysis. We set up an ANOVA design with the within-subject factor TASK (CC and IC) and tested for the interference contrast IC vs. CC. All statistical maps were thresholded at the voxel-level with *p* < 0.005 (uncorrected) and cluster-level corrected with the number of expected voxels per cluster (Köhler et al., 2016). To investigate the influence of PNC, *model 1* was estimated on data corrected for physiological noise as well as uncorrected data. *Model 2* and *model 3* were estimated on PNC data. To assess an overlap between activated brainstem clusters and LC location, we used the anatomical mask image in the MNI coordinate space based on Keren et al. (2009). This mask represents the extent of peak LC signal distribution, obtained from a sample of 44 healthy adults using high-resolution T1-weighted Turbo Spin Echo MRI.

We investigated the changes of luminance due to the presentation of color words of different length and ink color. In our lab outside the scanner, we used the toolbox phyphox^2^ (RWTH Aachen University, Aachen, Germany) running on a Samsung Galaxy tablet to record luminance during the Stroop task. Mean luminance changes during stimulus presentation were calculated to check for a difference of luminance between task conditions.

## Results

### Effect of Physiological Noise Correction on tSNR

**FIGURE 1:**
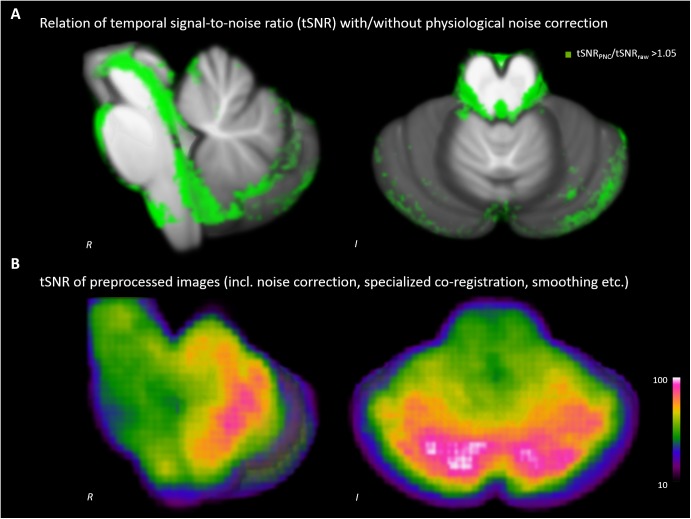
Impact of physiological noise correction (PNC) on temporal signal-to-noise ratio (tSNR). **(A)** Increase of tSNR due to PNC. **(B)** tSNR of the analyzed data after complete preprocessing including PNC, brainstem/cerebellum co-registration and smoothing etc.

We detected increases of the tSNR throughout the brainstem (see **Figure 1A**). The improvement was most pronounced around tissue borders to cerebral spinal fluid, but also within the midbrain, brainstem and cerebellum. The tSNR of our data used for analyses was 67.8 ± 15.9 on average within the SUIT template (**Figure 1B**).

### Effect of Physiological Noise Correction on Statistical Results (Stroop Interference Contrast)

**FIGURE 2:**
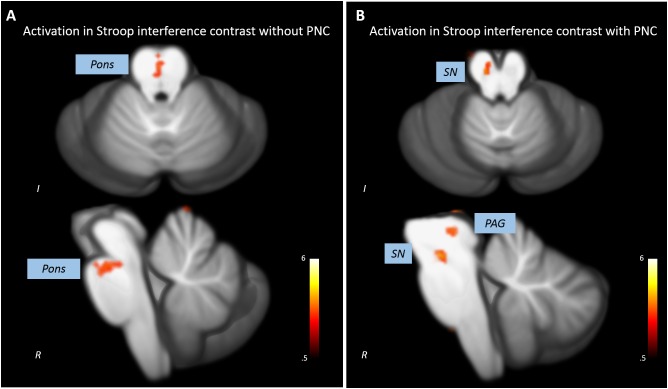
Impact of PNC on the statistical results revealed by the analysis of the Stroop interference contrast. **(A)** The analysis of uncorrected data leads to a statistical significant activation cluser in the upper pons and no midbrain/brainstem activations. **(B)** A significant activation in the substantia nigra (SN) and the periaqueductal gray (PAG) was detected when analyzing PNC data.

Without PNC, a cluster in the upper anterior pons showed increased activations in the incongruent compared to the congruent condition (*x* = 0, *y* = -24, *z* = -21, *t* = 3.69, *p* < 0.005, *k* = 12). Three clusters in the cerebellum also showed significant activation in the Stroop interference contrast (see **Figure 2A** and **Table 1**).

**Table 1:** Clusters with significant BOLD activations in the Stroop interference contrast with and without physiological noise correction of underlying data.

Region	Side	Cluster size	MNI coordinates			*t*
			*X*	*Y*	*Z*	
**Without physiological noise correction**		
Thalamus	R	17	18	-32	-1	3.8
Pons	R	16	0	-24	-21	3.69
Culmen	L	13	-2	-58	3	3.96
Dentate	L	20	-10	-48	-31	4.04
Anterior cerebellar lobe	R	12	14	-66	-33	4.55
**With physiological noise correction**		
Thalamus	R	69	11	-26	6	5.02
PAG	L	56	-7	-30	-4	3.89
SN	L	28	-6	-19	-16	4.29
Culmen	R	10	17	-48	-9	3.34
Nodule	L	16	-8	-49	-30	3.79
Cerebellar Tonsil	L	10	-25	-46	-44	3.41

After PNC, we found that three clusters in the midbrain were more activated in the incongruent than in the congruent condition (**Figure 2B**). One cluster most probably overlapped with the substantia nigra (SN, *x* = -6, *y* = -19, *z* = -16, *t* = 4.29, *p* < 0.005, *k* = 28) and one was situated adjacent to the aqueduct (periaqueductal gray, PAG, *x* = -7, *y* = -30, *z* = -4, *t* = 3.89, *p* < 0.005, *k* = 56). Additional activations were found in the cerebellum (see **Table 1**). There was no overlap of significant clusters of uncorrected and PNC data in the Stroop interference contrast.

### Behavioral and Physiological Reactions to the Stroop Task

Subjects responded significantly slower in incongruent (1223 ± 328 ms) than in congruent trials (1036 ± 216 ms, *t* = -4.16, *p* < 0.01), indicating a reliable induction of the Stroop effect. Errors and missing responses (*n* = 11) occurred in the IC only. Reaction times decreased with increasing trial number of the IC (*R*^2^ = 0.333, *p* < 0.05) and the CC (*R*^2^ = 0.349, *p* < 0.01) revealing a general learning effect.

**FIGURE 3:**
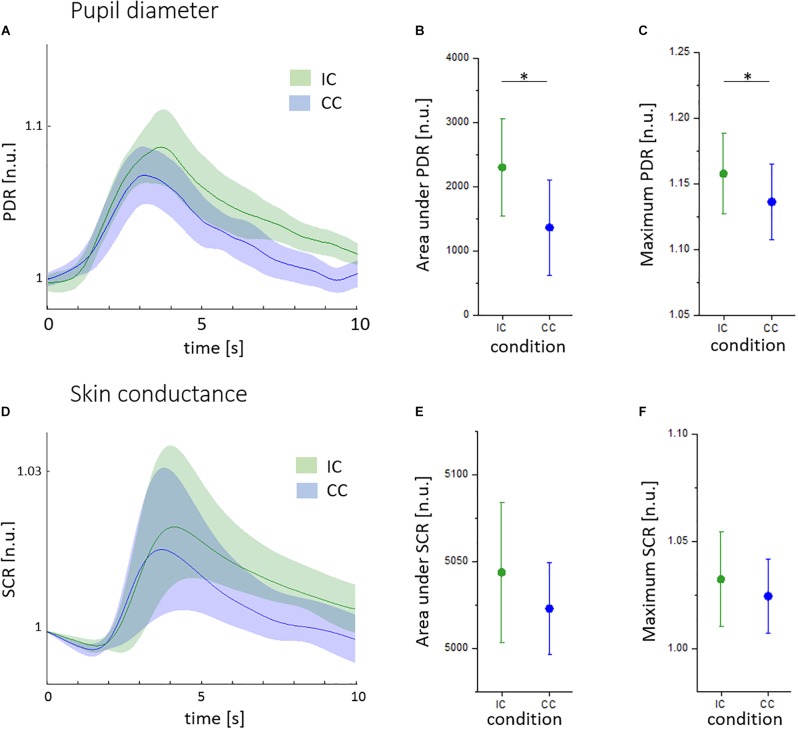
Pupillary and skin conductance reactions to the Stroop task given in normalized units (n.u.). **(A)** Mean pupil diameter reactions (PDR) during the congruent (CC, blue) and incongruent condition (IC, green), with standard deviation indicated by shaded areas. **(B)** Area under PDR during the CC (blue) and IC (green). **(C)** Maximum PDR during the CC (blue) and IC (green). **(D)** Mean skin conductance reactions in the CC (blue) and IC (green), with standard deviation indicated by shaded areas. **(E)** Area under SCR during the CC (blue) and IC (green). **(F)** Maximum SCR during the CC (blue) and IC (green). ^∗^*p* < 0.05.

As shown in **Figures 3A–C**, pupil diameter responses (PDR) were higher in incongruent (IC, green) than in congruent trials (CC, blue). The area under PDR was lower in the CC (1241 ± 1043 n.u.) compared to the IC (2155 ± 1110 n.u., *t* = -2.24, *p* < 0.05; **Figure 3B**). The maximum pupil size was 1.135 ± 0.038 n.u. in CC trails and 1.159 ± 0.041 n.u. in IC trials (*t* = -2.45, *p* < 0.05; **Figure 3C**). There was a significant habituation of pupillary responses during CC (R^2^ = 0.459, *p* < 0.01) and IC (R^2^ = 0.223, *p* < 0.05). The pupillary Stroop effect correlated to the Stroop interference effect (R^2^ = 0.464, *p* < 0.05).

There was no significant difference between SCR during the incongruent and congruent condition (**Figure 3D**). Similarly, the maximum, the area under SCR and the latency of SCR were not different (**Figures 3E,F**). We found no linear decrease of SCR indices over trials and no correlation to reaction times.

During the task, luminance decreased by 2.15 ± 1.45% in CC and 2.11 ± 1.44% in IC trials. There was no significant difference of luminance between the two conditions.

### SCR Parametric Modulation of Stroop Stimuli

Modulating Stroop stimuli using SCR, a significant activation of the SN in the Stroop interference contrast was replicated (*x* = -6, *y* = -24, *z* = -15, *t* = 3.91, *p* < 0.005, *k* = 39) together with most cerebellar clusters (see **Table 2**). In contrast, no activation of the periaqueductal gray was detected.

**Table 2:** Clusters with significant BOLD activations in the Stroop interference contrast after modulating task events by skin conductance responses (SCR) and pupil diameter responses (PDR) (with complete preprocessing).

Region	Side	Cluster size	MNI coordinates			*t*
			*X*	*Y*	*Z*	
**Parametric modulation with SCR**		
Thalamus	R	56	6	-24	8	4.7
Substania nigra	L	39	-13	-13	-12	3.91
Dentate	L	33	-16	-54	-28	4.5
Fastigium	L	23	-7	-49	-30	3.89
Anterior cerebellar lobe	R	42	5	-48	-33	5.94
**Parametric modulation with PDR**		
Thalamus	R	33	4	-18	8	3.17
Inferior colliculus	R	25	8	-38	-12	4.25
LC	L	39	-2	-36	-18	3.76
Medulla	L	23	-4	-37	-44	3.88
Culmen	L	10	-10	-62	-9	4.68
Culmen	R	13	6	-49	-10	3.27
Culmen	L	20	-19	-40	-18	4.38
Declive	R	19	29	-70	-20	4.83
Culmen	R	14	32	-54	-21	3.86
Cerebellar Lingual	R	13	8	-48	-24	3.38
Culmen	R	30	28	-40	-26	3.83
Culmen	R	15	11	-55	-27	4.22
Culmen	R	10	17	-42	-27	3.73
Declive of Vermis	R	22	0	-76	-27	3.47
Culmen	L	41	-12	-48	-28	4.57
Declive	L	10	-43	-68	-28	3.75
Declive	R	17	46	-54	-28	3.62
Declive	L	26	-30	-74	-28	3.56
Culmen	L	13	-40	-52	-30	4
Declive	R	45	42	-66	-30	3.83
Declive	R	10	11	-73	-30	3.72
Nodule	R	59	4	-56	-32	4.14
Uvula	R	13	8	-66	-36	3.49
Culmen	L	23	-42	-44	-38	4.48
Culmen	L	11	-28	-37	-38	3.86
Tuber	R	18	48	-60	-38	3.52
Pyramis	L	11	-12	-68	-38	3.39
Tuber	R	55	34	-74	-39	4.35
Tuber	L	13	-22	-85	-39	3.15
Pyramis	R	14	44	-70	-42	4.78
Uvula	L	26	-7	-66	-44	3.76
Cerebellar Tonsil	R	14	29	-62	-48	3.75
Cerebellar Tonsil	L	40	-32	-54	-50	6.08
Cerebellar Tonsil	R	12	12	-42	-50	3.41
Inferior Semi-Lunar Lobule	L	28	-30	-62	-52	3.47
Cerebellar Tonsil	R	44	5	-44	-54	3.91

*LC, locus coeruleus.*

### PDR Parametric Modulation of Stroop Stimuli

**FIGURE 4:**
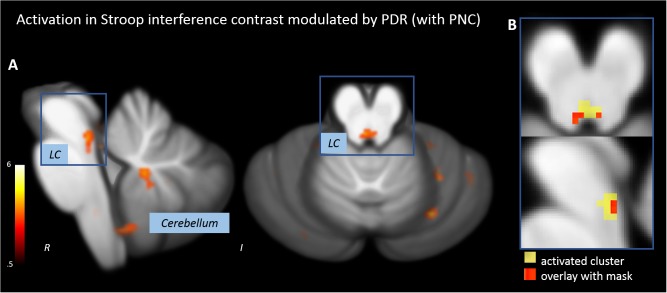
Activations in the Stroop interference contrast after modulating task events by pupillary responses. **(A)** Activation of an oblong cluster along the posterior border of the brainstem (locus coeruleus, LC). **(B)** Overlap of the activation cluster and a anatomical mask of the LC (Keren et al., 2009).

When Stroop stimuli were weighted by PDR, the Stroop interference contrast revealed a significant activation of a tube-like cluster at the posterior brainstem (**Figure 4A** and **Table 2**). The overlap with the anatomical mask of the LC suggested that the activated cluster overlaps with both lateral branches of the upper LC (**Figure 4B**).

## Discussion

In this study, we combined high-resolution fMRI, advanced brainstem co-registration, PNC and physiological recordings in order to precisely identify brainstem and midbrain regions involved in cognitive control. We demonstrate the enhancement of signal quality due to PNC as well as its beneficial effect on the statistical results. The signal to noise ratio was increased especially in tissue adjacent to arteries and spaces filled with cerebrospinal fluid. The analysis of PNC data replicated the involvement of the nigro-striatal dopaminergic system in cognitive control whereas uncorrected data revealed no brainstem/midbrain activations. We found that pupil size was a sensitive indicator of the level of cognitive demand. When Stroop stimuli were modulated by pupillary responses, we observed a significant activation of the LC.

About 20,000–50,000 neurons build the LC as a symmetric structure at the dorsal brainstem with a rostrocaudal extent of 12–17 mm and a diameter of about 2.5 mm (German et al., 1988; Fernandes et al., 2012). It begins rostrally in the caudolateral part of the mesencephalic central gray, at the level of the inferior colliculus, and extends caudally to a position in the lateral wall of the fourth ventricle (German et al., 1988; Köhler et al., 2016). Neurons of the LC project to various regions throughout the cerebral cortex, thalamus, hippocampus, brainstem, midbrain, cerebellum, and the spinal cord (Aston-Jones et al., 1999; Berridge and Waterhouse, 2003; Samuels and Szabadi, 2008a). Electrophysiological studies have shown that the LC selectively responds to salient stimuli that entail attention disruption and reorientation (Grant et al., 1988; Aston-Jones et al., 1999). The recorded LC activity was previously decomposed into a tonic and phasic component (Aston-Jones and Cohen, 2005). Tonic activity represents a kind of baseline arousal and is low during certain automatic behavior and drowsiness. LC neurons become phasically activated by salient stimuli (Rajkowski et al., 1994; Aston-Jones et al., 1999).

The LC is an important relay of the noradrenergic and dopaminergic circuitry of the brainstem (Fernandes et al., 2012). The LC-noradrenergic system regulates the balance between selective and flexible attention. At rest, we have recently shown that the LC is functionally integrated into the executive-control network (Bär et al., 2016). Under external stimulation, the LC seems to dynamically moderate the switch between distinct functional networks depending on the salience of incoming stimuli (Bouret and Sara, 2005; Corbetta et al., 2008). It has been demonstrated previously that the incongruent Stroop task condition, requiring inhibitory control, activates the neural cognitive control network consisting of the prefrontal cortex, the anterior cingulate cortex, the posterior parietal cortex and subcortical structures (Miller and Cohen, 2001; Critchley et al., 2005; Wagner et al., 2006). In our recent study, we additionally found that incongruent Stroop stimuli trigger the activation of the LC (Köhler et al., 2016). In the current study, we wanted to investigate the influence of enhanced preprocessing techniques on BOLD activations during the Stroop task.

Considering the spatial extent of brainstem nuclei, functional MR imaging requires highly precise approaches (Beissner et al., 2014). In our current analysis, the spatial resolution is far more sensitive compared to previous investigations (Murphy et al., 2014; Köhler et al., 2016). Additionally, we prepared the acquired functional data by PNC involving cardiac and respiratory signals. We demonstrated that the signal-to-noise ratio (SNR) of brainstem images benefits from PNC due to the presence of nearby major arteries and cerebrospinal fluid filled spaces (Brooks et al., 2013). Especially along the posterior border of the brainstem, an increase of temporal SNR was detected. In this region important nuclei of the ascending reticular system, the serotonergic system, the noradrenergic system etc. are located. After PNC and brainstem/cerebellum specialized co-registration an average temporal SNR of 67.8 was achieved. The SNR map resamples the results reported by Brooks et al. (2013). He highlighted the effect of noise from non-neural sources and reviewed different correction approaches (e.g., RETROICOR). In brainstem fMRI, the increase of signal quality by using RETROICOR was documented previously (see Harvey et al., 2008; Brooks et al., 2013). In our approach here, we use five respiration volume per time regressors additionally to the RETROICOR cardiac and respiratory phase regressors (Birn et al., 2008).

In this study, we show that PNC has a crucial impact on the results that revealed the analysis of BOLD activations during the Stroop task. Without PNC, the only activated cluster outside the cerebellum was located along the midline of the upper anterior pons. When analyzing PNC data, significant activations within the SN and a region adjacent to the aqueduct, presumably in the periaqueductal gray, were detected. The activation of the dopaminergic VTA and SN in the Stroop interference contrast was demonstrated previously (Köhler et al., 2016). The periaqueductal gray is an important interface of the midbrain/brainstem to cortical area with connections to areas involved in cognitive control such as the prefrontal and cingulate cortex as well as the ventral striatum (Wager et al., 2004; Faull and Pattinson, 2017). Via nigrostriatal and mesolimbic pathways, BOLD activations of the VTA/SN are associated with goal-directed motivational behavior (Amalric and Koob, 1993; Mannella et al., 2013). Therefore, the detected activations appear far more plausible when underlying data was corrected for the influence of physiological noise. As significant clusters in the Stroop interference contrast of corrected and uncorrected data did not overlap, PNC has fundamental influence on the results of our study. The analysis of uncorrected data revealed an activation running along the midline of the upper pons – an area susceptible to physiological noise. As our sample is rather small, statistical analyses are particularly vulnerable to spurious results due to artifacts and inaccurate co-registration. Thus, the correction for physiological noise seem essential to reveal adequate results.

In agreement with previous reports, the pupillary response (PDR) was sensitive to different demands of cognitive control in the Stroop task (Siegle et al., 2004; Laeng et al., 2011; Rondeel et al., 2015). Similar to reaction times, PDR decreased over the course of the experiment with a more pronounced decline in the congruent than in the IC. Thus, the learning effect during the task seems to be reflected in pupillary reactions.

Skin conductance responses were not different between both task conditions and were neither related to reaction times nor trial number. However, we observed a marked increase of SCR in the incongruent compared to the congruent condition, which might be statistically detectable in a bigger sample. The time course of skin conductance is determined by a tonic and a phasic component. Stimuli might elicit multiple overlapping SCR as well as an increase of the tonic level (Lim et al., 1997). Although the area under curve was demonstrated to capture SCR characteristics appropriately (Bach et al., 2010; Köhler et al., 2018a), the variance of SCR measures seems to be higher than pupillary indices.

The reactions of physiological signals were used to weight Stroop stimuli. Parametric modulation with SCR led to similar results as the analysis without modulation. In the brainstem/midbrain, the only activation was found in the SN. When events are combined with pupillary responses, a significant activation of a tube-like cluster at the posterior brainstem was found. The activated cluster overlapped with both lateral branches of the upper LC as indicated by the anatomical mask of the LC (Keren et al., 2009). Given the strong co-variation of LC activity during task performance and PD, we hypothesized that the pupillary signal might be useful to track LC activity (Rajkowski et al., 1994; Joshi et al., 2016; Eckstein et al., 2017). It seems that the modulation of Stroop events with physiological information was more accurately modeling the BOLD activation in the LC than the standard GLM.

However, the activated cluster is not completely matching the anatomical demarcation of the LC. The remaining activation between the two lateral branches of the LC might be due to a response of other nuclei located in this region, such as medial reticular nuclei or the dorsal raphe nucleus. Spatial smoothing might blur individual blobs of activation to a great cluster. However, smoothing improves the validity of statistical tests by normalizing the error distribution and compensating for small variations of individual brain anatomy.

Some limitations of our study have to be addressed. A drawback of our experimental design is the lack of luminance control as words presented on the screen vary in length and color. Because luminance was not different in both conditions, a systematic influence on our results seems unlikely. Furthermore, the statistical power is limited with respect to sample size and number of Stroop stimuli. The parametric modulator had a strong impact on our results, which might be due to the restricted statistical power. However, our results indicate that the statistical analysis benefits from a precise modulation of BOLD activation using physiological markers.

## Conclusion

In conclusion, we used a sophisticated fMRI technique and physiological recordings to investigate the involvement of brainstem/midbrain nuclei in cognitive control. We demonstrated a positive effect of PNC on data quality and statistical results. Finally, we corroborated previous findings that the dopaminergic SN and noradrenergic LC play a central role in cognitive control and demonstrated the reproducibility of this result on a small sample (Köhler et al., 2016). We investigated PD and skin conductance as autonomic markers of cognitive demand. By including the pupillary responses in our functional data analysis, we validated the location and functional role of the LC. We conclude that physiological signals are useful for modeling noise contaminating the BOLD signal but also BOLD signal changes of interest.

## Ethics Statement

This study was carried out in accordance with the recommendations of the Ethics Committee of the University Hospital Jena. The protocol was approved by the Ethics Committee of the University Hospital Jena. All subjects gave written informed consent in accordance with the Declaration of Helsinki.

## Author Contributions

AS conceived and designed the study, analyzed and interpreted the data, and prepared the manuscript. SK acquired the data and prepared the manuscript. FC analyzed the data and prepared the manuscript. GW conceived and designed the study, prepared the manuscript, and critically revised the manuscript. DG and JR critically revised the manuscript. K-JB conceived the study, prepared the manuscript, and critically revised the manuscript.

## Conflict of Interest Statement

The authors declare that the research was conducted in thound that three clusters in thee absence of any commercial or financial relationships that could be construed as a potential conflict of interest.

## Footnotes

1 http://www.fil.ion.ucl.ac.uk/spm

2 www.phyphox.org
